# Diet quality, sleep and quality of life in Parkinson’s disease: a cross-sectional study

**DOI:** 10.1007/s11845-022-03144-1

**Published:** 2022-09-02

**Authors:** Danielle Dunk, Philip Mulryan, Sean Affonso, Gerard W. O’Keeffe, Majella O’Keeffe, Aideen M. Sullivan

**Affiliations:** 1 Department of Nutritional Sciences, School of Life Course and Population Sciences, King’s College London, London, UK; 2 Department of Anatomy and Neuroscience, University College Cork, Cork, Ireland; 3 APC Microbiome Institute, University College Cork, Cork, Ireland; 4 School of Food and Nutritional Sciences, University College Cork, Cork, Ireland

**Keywords:** Dietary intake, Diet quality, Sleep, Parkinson’s disease, Quality of life

## Abstract

**Background:**

Parkinson’s disease (PD) is a neurodegenerative disorder characterised by motor and non-motor symptoms that impact quality of daily life, including diet and sleep. However, relatively little is known about dietary intake and quality in people with PD (PwP). Lifestyle factors, and how they relate to diet, are also insufficiently understood.

The aims of this study were to investigate dietary intake and quality, sleep and quality of life in PwP, and to explore the relationships between these factors.

**Methods:**

Forty-five community-dwelling participants with PD (*n* = 45) were recruited to this cross-sectional study through the Cork Parkinson’s Association, Ireland. Dietary intake was assessed using the EPIC food frequency questionnaire, and diet quality was assessed using the Healthy Diet Indicator. Dietary intakes were compared to Irish RDAs for adults > 65 years. Sleep duration and quality were subjectively measured using the PD Sleep Scale and Pittsburgh sleep quality index and objectively measured by actigraphy in a subset of participants (*n* = 27). QOL was measured using the validated PDQ-39 questionnaire.

**Results:**

Energy intake in PwP was significantly higher than that of the general population (2013 vs 1755 kcal/d, *p* = 0.01), despite their lower mean BMI (25.9 vs 27.7 kg/m^2^, *p* = 0.02). Intakes of carbohydrate, protein and fruits and vegetables were significantly higher in PwP compared to recommended and population intakes (all *p* < 0.01), but fibre intake was significantly lower than recommended (17.3 vs 25 g/d, *p* $\le$ 0.05). Seventy-eight percent of participants had poor dietary quality, and poor sleep quality was associated with poor QOL.

**Conclusions:**

Carbohydrates, protein, fruit and vegetable intakes were greater in PwP than population norms, but overall diet quality was low. Interventions to improve dietary and lifestyle factors may improve health and QOL in PwP.

## Introduction

Parkinson’s disease (PD) is a progressive neurodegenerative disorder affecting approximately 1% of the global population aged over 65 years [1]. Genetic and environmental risk factors are implicated in its aetiology [2]. Neuropathological hallmarks of PD include the progressive degeneration of midbrain dopaminergic neurons, which leads to loss of striatal dopamine levels and consequent motor impairments including rigidity, resting tremor and bradykinesia [2]. Additionally, intracellular accumulation of alpha-synuclein protein, in inclusions known as Lewy bodies that spread through the nervous system, contributes to non-motor PD symptoms including cognitive impairment, sleep disturbance, gastrointestinal dysfunction, dysphagia and depression [3]. Dopamine replacement pharmacotherapy is the mainstay treatment for PD, and clinical guidelines increasingly call for multi-disciplinary care for the management of motor and non-motor symptoms [4]. Side effects of long-term treatment, as well as natural disease progression, can lead to adverse consequences including impaired nutritional status and increased risk of malnutrition [5].

Unintended weight loss and reduced body mass index have been observed in PD cohorts, often despite increased energy intake [6]. The prevalence (0–24%) and risk (3–60%) of malnutrition amongst people with PD (PwP) varies, but aetiologically, symptoms such as constipation, dysphagia, olfactory dysfunction and appetite suppression can impact food intake, whilst involuntary motors symptoms increase energy expenditure, increasing the risk of malnutrition [5].

Studies investigating the impact of different dietary regimens on PD, notably the Mediterranean [7] and ketogenic diets [8] as well as bioactive compounds [9], are underway. However, an understanding of current dietary intake amongst PwP also warrants further research particularly given the conflicting data. Excessive carbohydrate intake amongst newly diagnosed PwP [10, 11], as well as a preference for sweet foods [12], has been reported. Data regarding protein intake is discordant; some studies have reported inadequate protein intakes [10, 12, 13], whilst others reported excessive protein intake compared to non-PD controls [13] and compared to recommended daily allowances [10]. Adherence to a protein redistribution diet, which is used to maximise absorption of levodopa, has also been shown to lead to low protein intake in this population [14]. However, there is some controversy regarding the data [15], and it has been reported that reduced protein intake may affect QOL [16].

Sleep disturbances occur in up to 98% of PwP, affecting both duration and quality of sleep as well as impacting daytime wakefulness [17]. In healthy populations, a bi-directional relationship between sleep quality and quantity, and diet, has been posited. Observational and experimental data indicate that inadequate sleep duration and quality adversely impact dietary intake [18, 19], and vice versa [20]. Inadequate sleep duration is associated with higher intakes of fat and protein [18, 21], whilst the impact on carbohydrate intake remains conflicted [22]. Additionally, overall dietary quality (DQ) is also adversely affected by sleep disturbances. Higher intakes of snacks, energy-dense foods [23, 24], confectionary [25] and sugar-sweetened drinks [24], as well as lower intakes of fruit, vegetables and wholegrains [26, 27], have been associated with inadequate sleep. A large observational study reported that poor sleep quality is significantly associated with greater energy intake and poor DQ, whilst good sleep quality significantly associated with Mediterranean diet adherence, which is indicative of better DQ [28]. Consequently, the preference for sweet foods, higher energy and lower protein intakes that are observed in PD patients may be influenced by sleep disturbances; therefore, the relationship between sleep and diet in PwP warrants further investigation. However, sleep studies should be interpreted with caution, as the majority are based on self-reported sleep and dietary intake and are thus open to response bias and misreporting. Additionally, controlled experimental and cross-sectional studies do not reflect habitual sleep, or the effects of chronic sleep debt.

Dietary intake and quality are associated with quality of life (QOL) [29], and, in PD, reduced QOL is commonplace [30]. Poor nutritional status and reduced QOL have been reported in PD [31], and a 6-week nutrition intervention for PwP at risk of, or experiencing, malnutrition significantly improved QOL [16]. In the Memory and Aging Project, a longitudinal cohort of elderly adults, the MIND dietary pattern (a combination of the dietary approaches used to prevent hypertension (DASH) and the Mediterranean diet) was shown to reduce the risk of developing PD, and to slow PD progression. The Mediterranean diet was observed to reduce the risk of disease progression, but no protective effect was observed with the DASH diet alone [32]. In other neurodegenerative disorders, positive correlations between greater intakes of fruit, vegetables and healthy fats (indicative of good DQ), and greater adherence to a Mediterranean diet, are associated with improved better QOL [31, 33, 34]. A systematic review found that better DQ or adherence to the Mediterranean diet in older adults with or at risk of chronic disease is positively associated with a better QOL [27]. Additionally, the MIND dietary pattern has been shown to be protective against risk of cognitive decline [35] and Alzheimer’s disease [36]. Given that PD is predominantly a disease of later life, these findings may be relevant to PwP, and they warrant further investigation.

As outlined, sleep disturbances and reduced QOL are prevalent in PD, and whilst little is known about diet intake and quality in PD, diet may affect QOL and, potentially, sleep. Understanding these interconnecting factors may inform tailored management strategies for PwP. Therefore, the aim of this study is to characterise dietary intake in a population of community-dwelling, free-living PwP in Ireland and to explore the relationship between dietary quality, sleep quality and QOL in this population.

## Methods

### Study design

A cross-sectional observational design was used to investigate DI, sleep quality and QOL in a free-living community population with PD. Associations between DI, sleep quality and QOL were explored. Ethical approval was granted by the Clinical Research Ethics Committee of the Cork Teaching Hospitals and University College Cork, Ireland.

### Sample

A convenience sample of *n* = 45 community-dwelling adults (aged ≥ 18 years) diagnosed with PD in accordance with the Queen Square London Brain Bank Criteria [37] was recruited between 2016 and 2018 from the Cork Parkinson’s Association, Ireland. Participants were provided with an information leaflet outlining details of the study, and informed consent was obtained from each participant. Participants under aged 18 years, those with PD diagnosis ≤ 6 months prior to study initiation, students, non-English speakers, pregnant women, prisoners, BMI ≤ 18 kg/m^2^ or unexplained weight loss (> 10% in the previous 6 months) were excluded. Participants who were illiterate or had cognitive impairment that would limit the ability to give informed consent were also excluded.

### Study variables

Demographic, anthropometric, dietary intake, sleep and QOL data were self-reported. Validated questionnaires were used. Participants completed hardcopies of the questionnaires during home and or clinic visits; questionnaires were self-completed, with assistance provided by the research team as needed. Data collection was carried out by a trained researcher within participants’ homes for approximately 45–60 min per visit.

### Dietary intake and quality

Dietary intake (DI) was determined using the European Prospective Investigation of Cancer (EPIC)-Norfolk Food Frequency Questionnaire (FFQ), a validated and widely used dietary assessment method [38] previously used in PD [10] which measures habitual food intake during the previous year. The full EPIC-FFQ methodology has been described elsewhere [38]. Briefly, the EPIC-FFQ has two sections: part one includes a list of 130 food items, of which usual consumption is indicated from nine frequencies (never or less than once/month to up to six times/day), part two includes additional questions to determine specific intakes of milk, cereal and fat. FFQs were analysed using FFQ EPIC and Nutrition Tool for Analysis (FETA, Windows, version 2.53) [39] to obtain daily EI, macronutrient and micronutrient data. If the frequencies of ≥ 10 food items were missing, the FFQ was considered incomplete and excluded from analysis. Analysed DI data was compared to the National Adult Nutrition Survey (NANS) (Irish Universities Nutrition Alliance (IUNA), 2011) and the RDAs for Ireland [40–42] and used to determine DQ. Mean dietary intakes amongst PwP were compared to Irish RDAs for adults > 65 years of age.

The Healthy Diet Index (HDI) was used to determine DQ as it was based upon nutritional recommendations closer to the population studied [43] and provided an objective analysis. Bertenez et al. [44] described recent updates to the original HDI [45] that represent current dietary guidelines for chronic disease. The HDI includes seven nutrients/food groups, each scored dichotomously with a value of 1 or 0 (Table 1). Dichotomous variables were summed to give an HDI score between 0 and 7; higher scores demonstrate better DQ.

**Table 1:** Healthy diet indicator (HDI) components and scoring criteria, as based on the WHO dietary guidelines for the prevention of chronic diseases [39]

Daily nutrient/food group intake	Scoring criteria for 1 point	Scoring criteria for 0 points
Saturated fat (% of TEI)*	$<$ 10	$\ge$ 10
Polyunsaturated fat (% of TEI)*	6–10	$<$ or $>$ 10
Cholesterol (mg)	$<$ 300	$\ge$ 300
Protein (% of TEI)*	10–15	$<$ 10 or $>$ 15
Fibre (g/d)	$>$ 25	$\le$ 25
Fruits and vegetables (g/d)	$\ge$ 400	$<$ 400
Free sugars^1^ (% of TEI)*	$<$ 10	$\ge$ 10

^*^% of total energy intake excluding alcohol

^1^Sum of glucose + maltose + sucrose from EPIC FFQ to estimate free sugars intake

### Sleep assessment

Sleep duration, latency and disturbance were objectively measured using actigraphy, a non-invasive, validated method for monitoring rest and activity cycles [46]. Participants wore an actigraph (Actigraph, Pensacola, FL, USA) on the non-dominant wrist for 7 days to track sleep and activity.

Sleep quality was also measured subjectively using three validated, self-report questionnaires: the Parkinson’s Disease Sleep Scale (PDSS), the PDSS-2 [47, 48] and the Pittsburgh Sleep Quality Index (PSQI) [49]. Participants with ^3^10% missing data were excluded from analysis.

The PDSS and PDSS-2 assess nocturnal symptoms associated with PD during the previous week. The questionnaires consist of 15 questions; each question is rated on a 10-point (PDSS) or 5-point Likert scale (PDSS-2). PDSS scores were collapsed into a 5-point Likert scale for analysis and to align with the more contemporary PDSS-2 scoring system. Scores for each question were summed ranging from 0 (no disturbance) to 60 (maximum disturbance) [48]. A score ≥ 18 suggests clinically relevant poor sleep quality [50].

The PSQI questionnaire measures sleep quality over the previous month and consists of 10 questions, nine completed by the participant and one by a bed partner/roommate. The PSQI has seven components: sleep quality, latency, duration, disturbances, medication, habitual sleep efficiency and daytime dysfunction, each scored between 0 and 3. Scores of participant-completed questions were summed to give a total score ranging 0–21. A score ≥ 5 suggests poor sleep quality [49].

### Quality of life

QOL was measured using the PD questionnaire-39 item (PDQ-39), a reliable and validated self-reported measure to assess health-related QOL over the previous month [51]. It consists of 39 questions assessing eight domains: mobility, emotional well-being, stigma, social support, cognition, communication, activities of daily living and bodily discomfort. Each question was scored on a 5-point Likert scale from 0 (never) to 4 (always). Scores for each question were summed to obtain a total PDQ-39 score; lower scores indicate better QOL. Domains were individually scored (sum of each item in the domain divided by maximum possible score within the domain/questions answered (for missing data)) ranging from 0 to 100. PDQ-39 summary index scores were calculated (sum of domain scores divided by eight) to provide a standardised QOL score [51].

### Statistical analysis

Descriptive statistics were used to characterise the population. For outcomes data, Shapiro–Wilk tests were used to determine normality in each variable analysed. A one-sample *t* test was used to compare DI data to RDAs and population intakes, and a paired samples *t* test was used to compare self-reported and objective sleep duration and latency. Pearson’s correlations were used to compare sleep quality, DQ and QOL scores of normally distributed variables; Spearman’s correlations were used for non-normally distributed variables. Correlations were unmatched pairs. Data are reported as means ± standard deviation (SD) unless otherwise specified. Statistical significance is reported as *p* < 0.05. SPSS (version 25) was used for statistical analysis.

## Results

### Participant characteristics

The sample consisted of *n* = 45 adults with a PD diagnosis. Twelve participants were removed due to incomplete dietary, sleep and/or QOL questionnaires; one participant was removed as an outlier. Thirty-three participants (19 male, 14 female) were included in the analysis; mean age was 69 $\pm$ 9 years, and mean BMI was 25.9 $\pm$ 3.9 kg/m^2^ (Table 2).

**Table 2:** Demographic and anthropometric data for free-living, community-dwelling patients with Parkinson’s disease (*n* = 33)

Measurement (units)	Males (*n* = 19)	Females (*n* = 14)	All (*n* = 33)
Age (years)	68 $\pm$ 9	71 $\pm$ 9	69 $\pm$ 9
Weight (kg)	78.2 $\pm$ 13.0	65.9 $\pm$ 9.7	73.7 $\pm$ 13.2
Height (m)	1.70 $\pm$ 0.10	1.65 $\pm$ 0.08	1.68 $\pm$ 0.09
BMI (kg/m^2^)	27.0 $\pm$ 4.2	23.9 $\pm$ 2.3	25.9 $\pm$ 3.9

Data reported as means ± standard deviation

### Dietary intake and quality

Mean DIs were compared to Irish RDAs for adults > 65 years of age (Table 3). EIs were similar to SACN (2011) recommendations; however, saturated fat, protein, salt, calcium, vitamin b12 and fruits and vegetable intakes were all significantly higher than the RDA, whilst fibre and vitamin D were significantly lower. Carbohydrate and fat intakes were within recommended ranges [36, 37]. There is no RDA for alcohol. Compared to general population intakes (IUNA, 2011) (Table 3), intakes of energy, carbohydrate, protein, salt and fruits and vegetables were significantly greater in PwP, whereas intake of vitamin D was significantly lower. Fibre, alcohol and vitamin B12 intakes were similar to the general population. No data were recorded for saturated fat in the NANS (IUNA, 2011). The majority of the participants had poor diet quality (*n* = 26). No difference in diet quality was observed between males and females (male 1.58 ± 1.1 min vs female 2.21 ± 0.9 min, *t*(− 1.804), *p* = 0.81).

**Table 3:** Dietary intake of people with Parkinson’s disease (*n* = 33) compared with age-appropriate recommended daily allowances (RDAs, Ireland) and age-matched intakes from the general Irish population intakes (NANS, 2011). Diet quality and diet quality tertile frequencies also reported for people with Parkinson’s disease

Dietary assessment	PwP (*n* = 33)	RDA	Difference		*p* value	Healthy population (*n* = 226)^4^	Difference	*p* value
**Daily dietary intake**								
Energy (kcal/d)^1^	2013 $\pm$ 502	2127	− 114		0.20	1755	+ 258	0.01*
Carbohydrate (% of TEI)^2^	48.8 $\pm$ 7.5	45–60	Within range		0.01**	44.1	+ 4.7	< 0.01*
Fat^^^ (% of TEI)^2^	34.6 $\pm$ 5.2	20–35	Within range		< 0.01**	34.7	− 0.1	0.89
Saturated fat (% of TEI)^2^	15.0 $\pm$ 4.1	< 10	+ 5		< 0.01*	-	-	-
Protein^^^^ (g/kgBW/d)^3^	1.22 $\pm$ 0.3	0.75	+ 0.47		< 0.01*	1.02	+ 0.2	< 0.01*
Fibre (g/d)^2^	17.3 $\pm$ 6.1	25	− 7.7		< 0.01*	18.9	− 1.6	0.14
Alcohol (g/d)^3^	5.3 $\pm$ 10.8	-	-		-	7.6	− 2.3	0.23
Salt (g/d)^3^	7.3 $\pm$ 2.3	6	+ 1.3		< 0.01*	6.3	+ 1	0.02*
Calcium (mg/d)^3^	962.0 $\pm$ 315.5	800	+ 162		0.01*	954	+ 8	0.89
Vitamin D (mg/d)^3^	3.4 $\pm$ 2.1	10	− 6.6		< 0.01*	6.9	− 3.5	< 0.01*
Vitamin B12 (mcg/d)^3^	6.8 $\pm$ 3.4	1.4	+ 5.4		< 0.01*	6.5	+ 0.3	0.56
Fruits and vegetables (g/d)^3^	499.4 $\pm$ 234.1	400	+ 99.4		0.02*	192	+ 307.4	< 0.01*
Free sugars (% of TEI)^3^	16.00 $\pm$ 4.5	< 10	+ 6		** < 0.01**	14.6	− 1.4	0.06
**Diet quality**								
	**Mean score**			**Score range**		**Frequency**		
HDI	2 $\pm$ 1			0–7				
Low DQ^5^ (***n***)						26		
Medium DQ^5^ (***n***)						5		
High DQ^5^ (***n***)						2		

*HDI* healthy diet index, *DQ* diet quality, *RDA* recommended dietary allowance, *TEI* total energy intake, *PwP* people living with Parkinson’s disease

Bolded data signifies statistical significance

Data as mean ± SD. Statistical significance *p* < 0.05

^1^SACN (2011)

^2^FSAI (1997)

^3^EFSA (2017)

^4^IUNA (2011)

^5^Low DQ = 0–2, Medium DQ = 3, High DQ = 4–7 (Berentzen, 2013)

^*^One-sample *t* test

^**^Based on mean RDA value. Carbohydrate: lower RDA value *p* = 0.01, upper RDA value *p* ≤ 0.01. Fat: lower RDA value *p* ≤ 0.01, upper RDA value *p* = 0.64, one sample *t* test

- no reported values available in the literature

^*n* = 32 ^^*n* = 29

### Sleep and quality of life

Sleep quality, sleep duration and QOL data are reported in Table 4. Mean sleep quality score, as assessed by PSQI, was 9 ± 5 and, as assessed by PDSS-2, was 22.2 ± 13, indicating that overall sleep quality was poor (^3^5 and ^3^18, PSQI and PDSS-2, respectively).

**Table 4:** Sleep quality, sleep duration and quality of life in people with Parkinson’s disease

Assessment tool	Mean scores	Score ranges
***Sleep quality***		
**PSQI (*n* = 28)**		
PSQI total	9 $\pm$ 5	0–21
Sleep quality	1 $\pm$ 1	0–3
Sleep latency	1 $\pm$ 1	0–3
Sleep duration	1 $\pm$ 1	0–3
Habitual sleep efficiency	2 $\pm$ 1	0–3
Sleep disturbance	2 $\pm$ 1	0–3
Use of sleep medication	1 $\pm$ 1	0–3
Daytime dysfunction	1 $\pm$ 1	0–3
Self-reported time taken to fall asleep^ (min)	25.85 $\pm$ 25.05	-
Self-reported total sleep time^ (min)	366.77 $\pm$ 80.86	-
**PDSS-2 (*n* = 30)**		
PDSS-2 total	22.2 $\pm$ 12.97	0–60
***Sleep duration***		
**Actigraphy (*n* = 27)**		
Latency (min)	11.22 $\pm$ 9.44	-
Total sleep time (min)	452.22 $\pm$ 81.18	-
Wake after sleep onset (min)	64.85 $\pm$ 5.76	-
Number of awakenings	10 $\pm$ 6	-
Average awakening (min)	6.81 $\pm$ 2.72	-
***Quality of life***		
**PDQ-39 (*n* = 30)**		
Total	42 $\pm$ 26	0–156
Mobility	36.82 $\pm$ 27.27	0–100
Activities of daily living	31.25 $\pm$ 27.13	0–100
Emotional well-being	20.83 $\pm$ 18.86	0–100
Stigma	12.29 $\pm$ 15.26	0–100
Social support	9.44 $\pm$ 14.76	0–100
Cognition	35.07 $\pm$ 19.52	0–100
Communication	20.69 $\pm$ 22.70	0–100
Bodily discomfort	35.83 $\pm$ 27.70	0–100
Summary index	25.28 $\pm$ 15.17	0–100

*PSQI* Pittsburgh sleep quality index, *PDSS-2* Parkinson’s disease sleep scale 2, *PDQ39* Parkinson’s disease questionnaire 39 item

Data as mean ± SD. ^*n* = 31

PSQI score > 5 = poor sleep quality, PDSS2 score > 18 = poor sleep quality, PDQ39 higher score = worse quality of life

Objective sleep duration, measured by actigraphy (~ 7.5 $\pm$ 1.4 h), was in line with current recommendations (7–8 h) [52]. However, self-reported sleep duration (~ 6.0 $\pm$ 1.4 h) was significantly lower than objective sleep duration (*t* =  − 4.54, *p* ≤ 0.01). Similarly, self-reported sleep latency (24.77 $\pm$ 26.10 min) was significantly greater than objectively recorded sleep latency (11.08 $\pm$ 9.60 min) (*t* = 2.61, *p* < 0.02) (Table 5). There were no significant sex differences in sleep duration (PSQI-derived sleep duration: male 371 ± 79.9 min vs female 364.1 ± 92.9 min, *t*(0.203), *p* = 0.841; actigraphy sleep duration: male 463.1 ± 74.2 min vs female 97.2 ± 29.3 min, *t*(0.639), *p* = *0*.495). There were no significant sex differences in sleep latency (PSQI-derived sleep latency: male 28 ± 30.7 min vs female 19.9 ± 14.3 min, *t*(0.822), *p* = 0.419; actigraphy sleep latency: male 9.8 ± 9 min vs female 12.8 ± 10.6 min, *t*(− 0.786), *p* = 0.439).

**Table 5:** Objective (actigraphy) versus subjective (self-report) sleep latency and sleep duration in people with Parkinson’s disease (*n* = 26)

Sleep assessment (*n* = 26)	Mean number of minutes	*p* value
**Latency**		
Subjective^1^	24.77 $\pm$ 26.10	-
Objective^2^	11.08 $\pm$ 9.60	-
Difference	13.69 $\pm$ 26.75	0.02*
**Duration**		
Subjective^1^	368.08 $\pm$ 83.92	-
Objective^2^	453.23 $\pm$ 83.63	-
Difference	− 85.15 $\pm$ 95.70	< 0.01*

Data reported as means ± SD

^1^Self-reported in PSQI

^2^Measured using actigraphy

^*^Statistically significant at *p* < 0.05, paired samples *t* test

Table 6 shows the associations between sleep quality, DQ and QOL using Spearman’s and Pearson’s correlation where appropriate. No correlation was found between sleep quality assessed by PDSS-2 or PSQI and DQ. PDSS-2 and PSQI total score showed no significant association between sleep quality and DQ (*r* =  − 0.260, *p* = 0.18 and *r*s =  − 0.229, *p* = 0.22 respectively). Similarly, there was no significant association between QOL, as measured by PDQ-39 total score or the summary index, and DQ (*r* =  − 0.122, *p* = 0.52 and *r* =  − 0.152, *p* = 0.42 respectively). A significant moderate positive correlation was found between both measures of sleep quality, PDSS-2 and PSQI total scores (*rs* = 0.455, *p* = 0.02).

**Table 6:** Correlations between sleep quality, diet quality and quality of life scores in a population of people with Parkinson’s disease

	**Diet quality**		**Sleep quality**			
**Assessment tool**	HDI total		PSQI total score		PDSS-2 total score	
	*r/r*_*s*_	*p* value	*r/r*_*s*_	*p* value	*r/r*_*s*_	*p* value
**Sleep quality**						
PSQI total score	− 0.260^2^	0.18	-	-	-	-
Sleep quality	− 0.90^1^	0.65	-	-	-	-
Sleep latency	− 0.063^1^	0.75	-	-	-	-
Sleep duration	− 0.006^1^	0.98	-	-	-	-
Habitual sleep efficiency	− 0.207^1^	0.29	-	-	-	-
Sleep disturbance	− 0.206^1^	0.29	-	-	-	-
Use of sleep medication	− 0.230^1^	0.24	-	-	-	-
Daytime dysfunction	− 0.184^1^	0.35	-	-	-	-
PDSS-2 total score	− 0.229^1^	0.22	0.455^1^	0.02*	-	-
**PDQ-39 (QOL)**						
Total	− 0.122^2^	0.52	0.350^2^	0.08	0.499^1^	0.01*
Mobility	− 0.143^2^	0.45	0.060^2^	0.77	0.283^1^	0.14
Activities of daily living	− 0.142^1^	0.45	0.181^1^	0.38	0.463^1^	0.01*
Emotional well-being	0.069^1^	0.72	0.447^1^	0.02*	0.433^1^	0.02*
Stigma	− 0.030^1^	0.87	0.118^1^	0.57	0.114^1^	0.56
Social support	− 0.197^1^	0.30	0.115^1^	0.58	0.152^1^	0.44
Cognition	− 0.050	0.79	0.429^2^	0.03*	0.490^1^	0.01*
Communication	− 0.282^1^	0.13	0.249^1^	0.22	0.259^1^	0.18
Bodily discomfort	− 0.158^2^	0.41	0.417^2^	0.03*	0.635^1^	< 0.01*
Summary index	− 0.152^2^	0.42	0.432^2^	0.03*	0.532^1^	< 0.01*

*PSQI* Pittsburgh Sleep Quality Index, *PDSS-2* Parkinson’s Disease Sleep Scale 2, *PDQ-39* Parkinson’s Disease Questionnaire 39 item

Bolded data signifies statistical significance

^1^Spearman’s correlation

^2^Pearson’s correlation

^*^Statistically significant at *p* < 0.05

A significant positive association was found between QOL, as measured by PDQ-39 summary index and sleep quality using both PDSS-2 and PSQI total score (*r* = 0.432, *p* = 0.03 and *rs* = 0.532, *p* ≤ 0.01 respectively). Similar correlations were reported when the PDQ-39 total score and PDSS-2 total score were used (*rs* = 0.499, *p* = 0.01). There was an insignificant trend towards a moderate positive association between PDQ-39 and PSQI total score (*r* = 0.350, *p* = 0.08).

Analysis of individual domains of QOL that compose the PDQ-39 found significant moderate positive associations between the emotional well-being (*rs* = 0.433, *p* = 0.02 and *rs* = 0.447, *p* = 0.02), cognition (*rs* = 0.490, *p* = 0.01 and *r* = 0.429, *p* = 0.03) and bodily discomfort (*rs* = 0.635, *p* ≤ 0.01 and *r* = 0.417, *p* = 0.03) domains and sleep quality as measured by PDSS-2 and PSQI, respectively. A significant moderate positive association was also found between activities of daily living and PDSS-2 (*rs* = 0.463, *p* = 0.01).

## Discussion

This study aimed to characterise DI in a population of community-dwelling, free-living people with PD in Ireland, and to explore the relationships between DQ, sleep quality and QOL. Significant differences were observed between DIs in PwP compared to RDAs for Ireland and for the general population of the same age. The PD population had low DQ overall and significantly higher intakes of energy, carbohydrates, and fruit and vegetables, compared to the general population, whilst showing significantly lower fibre intake. Contradictions between carbohydrate, fruits and vegetables and fibre intake could be due to misreporting in the FFQ or to the small sample size. The free sugar intake in PwP was significantly higher than the RDA, whilst vitamin D intake was significantly lower, highlighting the potential need for vitamin D supplementation in this cohort. Consistent with current literature [53, 54], sleep quality was clinically poor as identified by two measures of sleep quality, PSQI and PDSS-2. Habitual sleep efficiency and sleep disturbance were the most affected domains as assessed on the PSQI. QOL was moderate in this cohort of PwP, with mobility, activities of daily living, cognition and bodily discomfort being the most affected domains.

Contrary to previous studies on healthy populations [26, 28, 29], DQ was not found to be associated with sleep quality or QOL in PD. However, poor sleep quality was significantly associated with reduced QOL, particularly in activities of daily living, emotional well-being, cognition and bodily discomfort domains.

Energy intake was similar to SACN (2011) recommendations, but significantly higher than population intakes (2127 vs 1755 kcal) despite a lower mean BMI amongst PwP (25.9 kg/m^2^) compared to the mean population BMI (27.7 kg/m^2^) (IUNA, 2011). This is consistent with existing literature [13, 55] and could be due to an increase in EE in PD, related to motor symptoms and treatment complications, suggesting that EIs are unable to compensate for increased requirements. However, Marczewska and colleagues reported no significant difference in EI or BMI in PwP compared to spouse controls, although it is expected that intakes of PwP and spouses would be similar [10]. In this population of PD patients, mean daily protein intake (1.22 g/kg/d) was significantly higher than the RDA (0.75 g/kg) and population intakes (1.02 g/kg), which is consistent with the research of Barichella et al. (2017) and Marczewska et al. (2006), both of whom also reported significant positive associations between protein intake, disease duration and higher levodopa doses [10, 13]. Protein-restricted diets have been proposed to reduce progression of PD and to affect the therapeutic efficiency of levodopa [15]. Barichella and colleagues [13] reported that protein intake of 10 g in excess of the RDA is associated with increased levodopa dose. Whilst no medication data is available for the participants in our study, future research should consider whether intakes such as that reported (1.22 g/kg/d) affect medication use and disease progression.

The percentage of energy intake from carbohydrate was significantly higher in this cohort of PwP than in the healthy population, though it was within the RDA, which supports previous research by Ådén et al. (2011) and Marczewska et al. (2006) [10, 11]. Estimated intake of free sugars was significantly higher than the recommended intake. This could potentially be related to the preference for sweet foods by PwP, as reported by Aiello et al., (2015) although it should be noted that unvalidated questionnaires were used in that study [12]. Preference for sweet food is theorised to be due to excessive dopamine neurotransmission related to PD treatment [56], although data from human studies is lacking.

In PwP, the percentage of energy obtained from fat intake was also within the RDA and similar to population intakes. This is consistent with dietary fat intake, assessed using the EPIC FFQ, from a cross-sectional study of 45 Italian PwP and their spouses [10]. In contrast, Barichella (2017) reported intakes of higher fat and saturated fat in PwP compared to controls. These differences in the literature could be due to the different FFQs used to measure DI. In the current study, the percentage of energy from saturated fat and free sugar intake was significantly higher than recommended, and fibre intake was lower than recommended; both of these are indicative of poor dietary quality. Indeed, 78% of participants were found to have poor overall DQ. Currently there is a lack of research exploring DQ in PD, but these findings suggest that there may be an opportunity for dietary management strategies to improve DQ in this cohort of patients.

Limitations of this study are recognised and include the small sample size which limits generalisability, the cross-sectional nature of the data which does not reflect longitudinal changes and the absence of data on medication use. Further, the EPIC FFQ was originally developed for the prospective investigation in cancer trial but, despite this, has been used previously in PD cohorts [10]. FFQ approaches for dietary assessment are regularly used in studies with PwP; however, shorter validated tools would be a welcome addition to the field, particularly given the potential impact of PD symptoms on the completion of longer FFQ which commonly contain in excess of 100 food items. Whilst the limitations of the existing data are acknowledged, this is the first study to investigate the relationship between DQ, QOL and sleep quality in a cohort of PD patients. It found no correlation between DQ and sleep quality, as assessed by two independent measures, PDSS-2 and PSQI. Evidence from previous studies on populations of healthy adults showed that poor sleep quality is significantly associated with lower DQ [57, 58]. However, these studies did not use validated tools to measure sleep quality, and so the results should be interpretated with that consideration. This study should encourage further work in the area, particularly research focused on individuals living well in the community, and interventions to address lifestyle factors to improve overall health and QOL in PwP.

## Abbreviations

BMI: Body mass index
DI: Dietary intake
DQS: Dietary quality
DQS: Dietary quality score
EE: Energy expenditure
EI: Energy intake
EFSA: European Food Safety Authority
EPIC: European Prospective Investigation of Cancer
FETA: FFQ EPIC and Nutrition Tool for Analysis
FFQ: Food Frequency Questionnaire
FSAI: Food Safety Authority of Ireland
HDI: Healthy Diet Indicator
IUNA: Irish Universities Nutrition Alliance
NANS: National Adult Nutrition Survey
PD: Parkinson’s disease
PDSS: Parkinson’s disease Sleep Scale
PDSS-2: Parkinson’s disease Sleep Scale-2
PDQ-39: Parkinson’s disease questionnaire-39 item
PSQI: Pittsburgh Sleep Quality Index
PwP: People with Parkinson’s disease
QOL: Quality of life
RDA: Recommended daily allowance
SACN: Scientific Advisory Committee on Nutrition
WHO: World Health Organisation
